# Supplementary material for: Molecular mechanisms underlying the early steps of floral initiation in seasonal flowering genotypes of cultivated strawberry
Source: Front Plant Sci. 2025 Jun 19;16:1563658. doi: 10.3389/fpls.2025.1563658 (PMC12222167; doi:10.3389/fpls.2025.1563658)
Supplement: Supplementary file 1 [file DataSheet1.docx]

## Supplemental Material


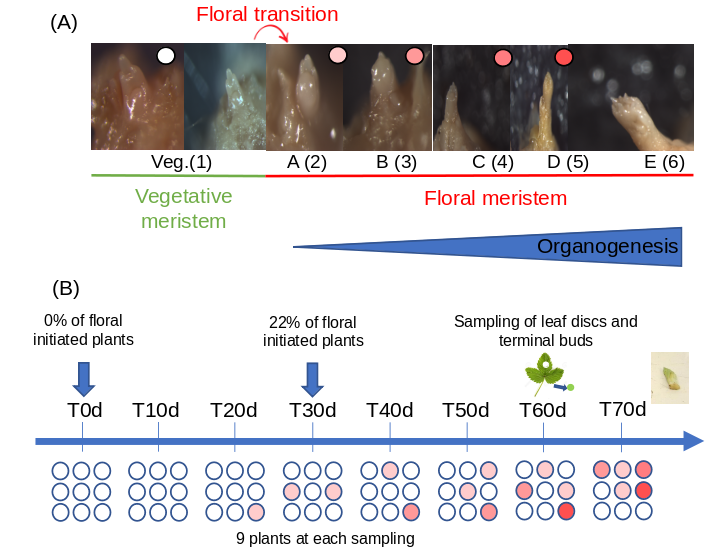


###### **Supplemental Figure S1**: RNA-Seq sampling protocol. (A) Changes in the stage of the terminal meristem over time (modified from Gaston et al., 2021). At the vegetative stage (Veg., numbered 1) the apical dome of the meristem is enclosed or partially enclosed in the developing stipule of the youngest leaf. The subsequent initiation stages of the inflorescence from A to E are numbered 2–6. A: Apical dome rises above the developing stipule. B: First bract primordium. C: Four distinct sepal primordia. D: Growth of sepal and petal. Meristem stages adapted from Jahn and Dana (1970) and Taylor et al. (1997). (B) Hypothetical example of a set of nine plants for which the stage of the terminal meristem was identified after regular sampling. Leaf and terminal bud samples were collected when 0% and 20–25% of plants showed evidence of floral initiation. We assumed that the early events of floral initiation would be captured between these time points.


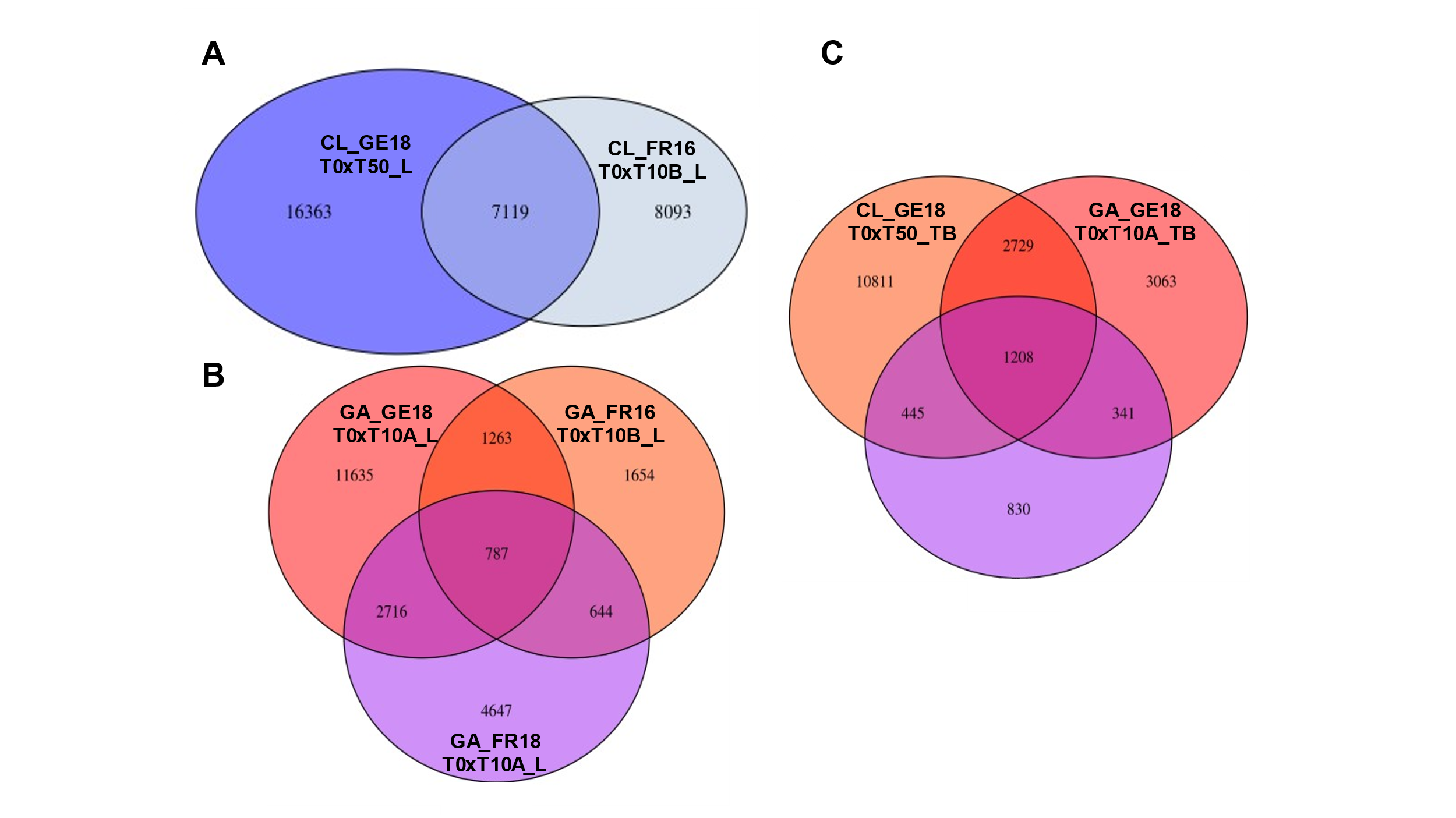


###### **Supplemental Figure S2**: Venn diagrams showing the intersection of genotypes in specific temporal and geographical contexts. (A) The blue region represents cultivar Clery (CL) in Germany w29–35 (CL_GE T0xT50 L), whereas the light blue region represents CL in France 2016 w29–33 (CL_FR_2016 T0xT10 L). (B) The red region represents cultivar Gariguette (GA) in Germany w29–32 (GA_GE T0xT10 L), the orange region signifies GA in France 2016 w29–33 (GA_FR T0xT10 L), and the purple region indicates cultivar GA in France 2018 w29–32 (GA_FR_2018 T0xT10 L), all for both leaf and terminal bud tissues. (C) The orange region represents cultivar CL in Germany w29–35 for terminal bud tissue (CL_GE T0xT50 TB), the red region signifies cultivar GA in Germany w29–32 for terminal bud tissue (GA_GE T0xT10 TB), and the purple region indicates cultivar GA in France 2018 w29–32 for terminal bud tissue (GA_FR_2018 T0xT10 TB).


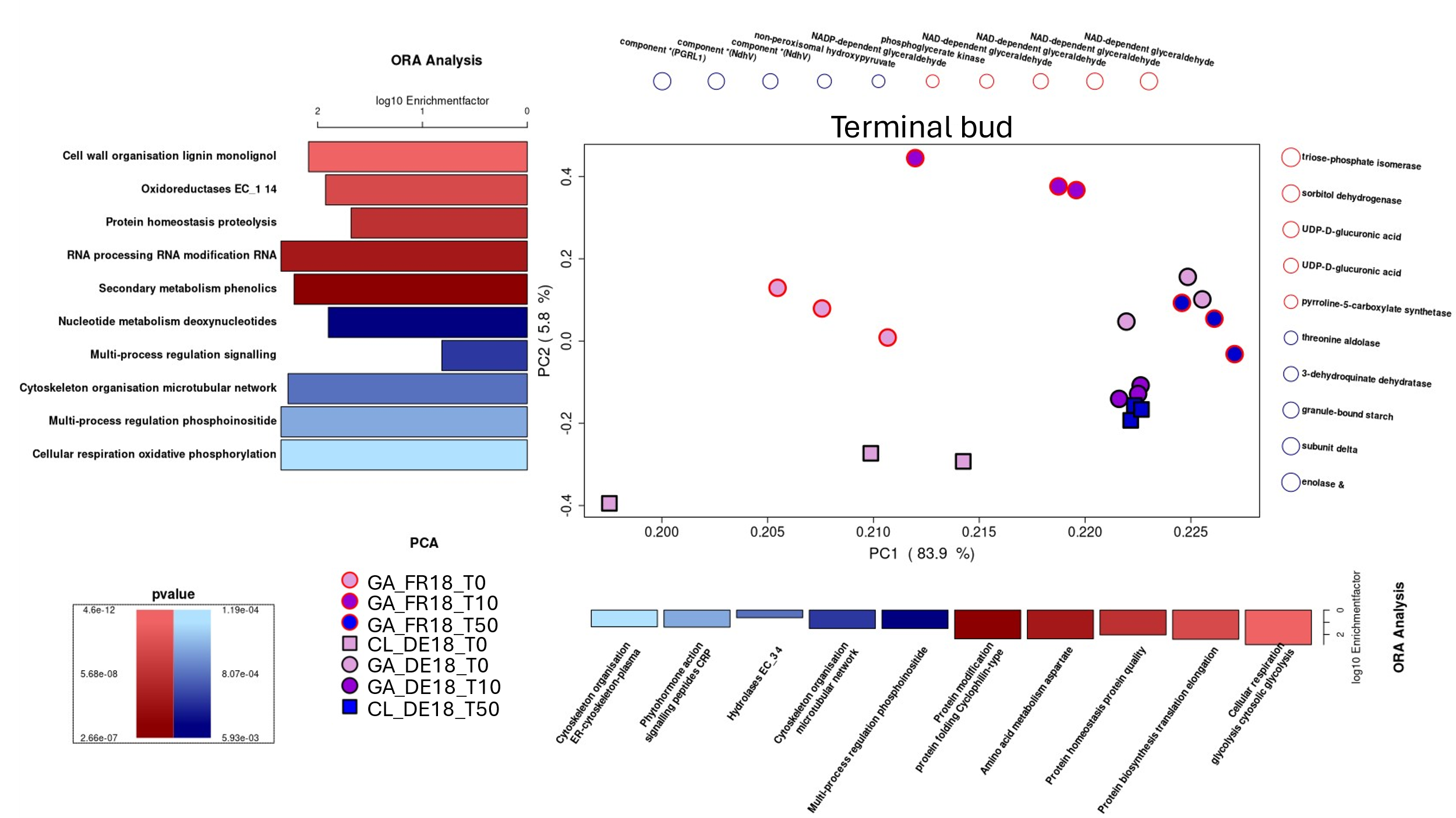


###### **Supplemental Figure S3**: Principal component analysis (PCA) applied to differentially expressed genes in the terminal buds of Clery (CL) and Gariguette (GA) cultivars from France in 2018 (FR_2018) and Germany (GE) at w29 (T0), w32 (T10) and w35 (T50). Overrepresentation analysis (ORA) was then applied to the MapMan annotation of protein classes based on the loadings of principal components 1 (PC1) and 2 (PC2). The top 5 MapMan bincodes resulting from ORA (p < 0.01) and their involvement in the separation along PC1 and PC2 in positive (red) and negative (blue) directions, are illustrated as bar plots of the log_10_ enrichment factor. Genes highly implicated in the positive (red) and negative (blue) separation of PCA are depicted above (PC1) and to the right (PC2).
